# Gastric bypass with unknown intestinal malrotation: Required attitude^☆^

**DOI:** 10.1016/j.ijscr.2013.09.012

**Published:** 2013-09-25

**Authors:** Radwan Kassir, Pierre Blanc, François Varlet, Christophe Breton, Patrice Lointier

**Affiliations:** a Department of Digestive Surgery, Clinique Chirurgicale Mutualiste, Saint Etienne, France; b Department of Pediatric Surgery, CHU Hospital, Jean Monnet University, Saint Etienne, France; c Department of Digestive Surgery, Clinique de la Châtaigneraie, France

**Keywords:** Obesity, Gastric bypass, Common mesentery, Laparoscopy, Intestinal malrotation, Surgical technique

## Abstract

**INTRODUCTION:**

Intestinal malrotations are rare and may be asymptomatic until adulthood. There are only a few descriptions of gastric bypass with intestinal malrotation. If the duodenojejunal angle is not correctly seen, as is generally the case, there is a risk of creating an antiperistaltic anastomosis.

**PRESENTATION OF CASE:**

We describe required attitude and cases of gastric bypass performed on two patients who had a complete common mesentery. In both of our patients, the transverse colon was not running across the abdomen and the duodenojejunal angle was “absent”. We therefore looked for the caecum in order to unravel all of the small intestine. We were able to carry out Roux en Y gas- 42 tric bypass with uncomplicated post-operative courses for both 43 patients. The result in weight loss was perfect and identical to that of patients without anatomical abnormality.

**DISCUSSION:**

In our cases, ultrasound to investigate for gallstones did not provide a pre-operative diagnosis. It is extremely difficult to investigate for the mesenteric vessels by ultrasound in obese patients and for this reason the finding was not made preoperatively. The most important thing to do is make the diagnosis of malrotation preoperatively. For this reason the golden rule in performing a gastric bypass is to clearly visualise the duodenojejunal angle which allows an unknown bowel malrotation to be identified. Following these cases, the study of the oeso-gastro-jejeunal transit is now part of our pre-operative assessment.

**CONCLUSION:**

Bariatric surgeons need to be aware of these abnormalities. If a common mesentery is present the gastric bypass can still be performed.

## 1 Introduction

Intestinal malrotations are rare and may be asymptomatic until adulthood. We describe cases of gastric bypass performed on two patients who had a complete common mesentery. If the duodenojejunal angle is not correctly seen, as is generally the case, there is a risk of creating an antiperistaltic anastomosis.

## 2 Presentation of case

Two patients were admitted for a gastric bypass for obesity (BMI 41 kg/m^2^, no other history and the decision to operate was made in a multidisciplinary meeting). Regardless of the type of gastric bypass (Roux en Y or omega), we always begin the procedure by identifying the angle of the Treitz ligament in order to confirm that the gastric bypass can be carried out (looking for example for adhesions or a paraduodenal hernia). In both of our patients, the transverse colon was not running across the abdomen and the duodenojejunal angle was “absent”. We therefore looked for the caecum in order to unravel all of the small intestine. This was located in the left iliac fossa, and therefore we suspected intestinal malrotation. We then found the duodenojejunal angle in the right hypochondrium. The two operations were done par the same surgeon. We were able to carry out Roux en Y gastric bypass with uncomplicated post-operative courses for both patients.

## 3 Discussion

**Fig. 1:**
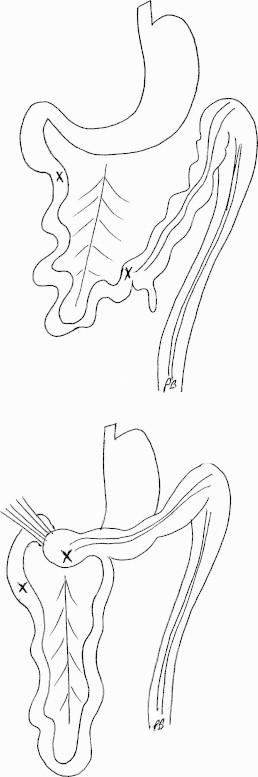
Rotation abnormalities. *Above*: complete common mesentery and *below*: incomplete common mesentery.

Only a few descriptions of gastric bypass with intestinal malrotation are available and these are limited to clinical case reports.^1–9^ During embryonic development, the intestine undergoes three 90° anti-clockwise rotations around the axis formed by the superior mesenteric artery.^10^ These rotations occur simultaneously with the small bowel's reintegration into the abdomen. After this, peritoneal fixation occurs between the end of the 12th week of development and birth. A malrotation describes rotation and fixation abnormalities with an abnormal relationship between the duodenojejunal angle and the ileocaecal valve, with abnormal colonic attachments. The complete common mesentery occurs when rotation stops at 90°: the small bowel is located entirely on the right of the abdomen, the duodenojejunal angle is not clearly apparent and is located to the right of the mesenteric vessels and the caecum is located in the left iliac fossa (Fig. 1).^10^ The small bowel mesentery and colonic mesentery are therefore continuous in the same plane. In France the prevalence of intestinal malrotations is reported to be in the region of 1/10,000–1/20,000 births. Most occur before the age of 1 with a peak incidence during the first month after birth (64–80% of cases). The risk falls significantly beyond 1 year of age (9–18% of cases). Other rotation abnormalities such as total absence of rotation, inverse rotations and over-rotations are even rarer. The prevalence of small intestine volvulus in adults due to an intestinal rotation abnormality is not known, although it appears to be extremely rare as fewer than 100 cases have been published. The fact that small intestine volvulus in adulthood is exceedingly rare means that it is a poorly understood disorder which may not be recognised by surgeons, and thus creates a risk of pre-operative diagnostic error.

**Fig. 2:**
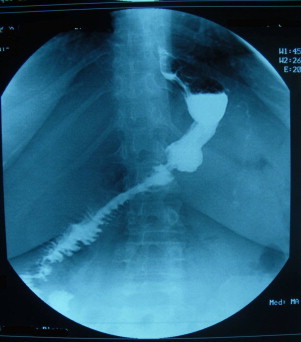
post-operative oeso-gastro-duodenal transit showing the gastrojejunal anastomosis.

**Fig. 3:**
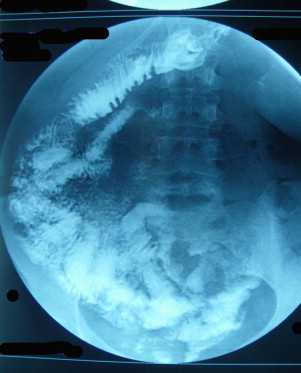
post-operative oeso-gastro-duodenal transit: appearances showing a complete.common mesentery with all of the small intestine loops shifted to the right of the midline. The duodenojejunal angle is not evident and is placed on the right.

**Fig. 4:**
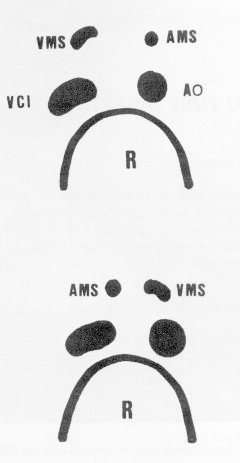
Position of the mesenteric vessels in a transverse section beneath the pancreas. *Above* normal appearance: the superior mesenteric artery lies in front of the aorta and the superior mesenteric vein lies in the front of the inferior vena cava. *Below*, vascular malposition, the artery is in front of the vena cava and the vein is in front of the aorta.

These rotation abnormalities may be suspected in adults as a result of ultrasound, upper gastrointestinal studies or computed tomography (Figs. 2–4),^3,10,11^ though these investigations are not routinely performed before bariatric surgery. In our cases, ultrasound to investigate for gallstones did not provide a pre-operative diagnosis. It is extremely difficult to investigate for the mesenteric vessels by ultrasound in obese patients and for this reason the finding was not made preoperatively. This is often the case in the literature reports.^5^ Following these cases, the study of the oeso-gastro-jejeunal transit is now part of our pre-operative assessment so that we can screen for an abnormality of the angle of the oesophagus and cardia as well as looking for malrotation, which is a recent advance (Figs. 2 and 3).

In gastric bypass, it is highly recommended that surgeons check that no abnormalities are present in the angle of the Treitz ligament before dividing the stomach. This is a routine procedure for us^12^ and led us to suspect a malrotation. The anatomical abnormalities which should alert the surgeon are a duodenojejunal angle on the right of the mesenteric vessels, absence of a transverse colon across the abdomen, the small intestine on the right side of the abdomen and the caecum on the left (Fig. 1). If uncertain, the whole small intestine should be unravelled in order to identify both ends. The “incomplete” Ladd procedure can help to understand the anatomy.^13^ This manoeuvre, described initially to treat volvulus due to small intestinal malrotation, consists of five operative stages: checking the small intestine, releasing the caecum, releasing the duodenojejunal angle, appendicectomy and positioning as a complete common mesentery (i.e. placing all of the small intestine in the right half of the abdomen, beginning with the first jejunal loop which is placed as far as outwards as possible beneath the liver and retracting the caecum towards the left iliac fossa as low as possible).^10^ This manoeuvre allows the whole anatomy to be checked. Routine appendicectomy was not performed in the published cases.

**Fig. 5:**
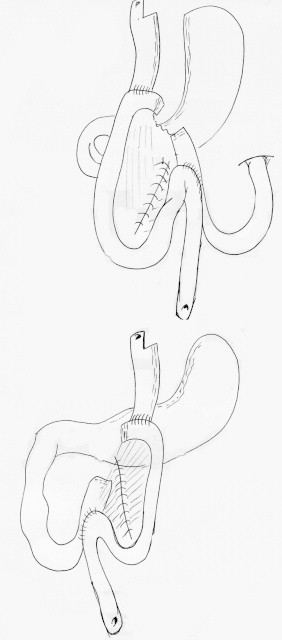
Roux en Y gastric bypass on loop: *above* with normal anatomy, *below* with a complete common mesentery.

**Fig. 6:**
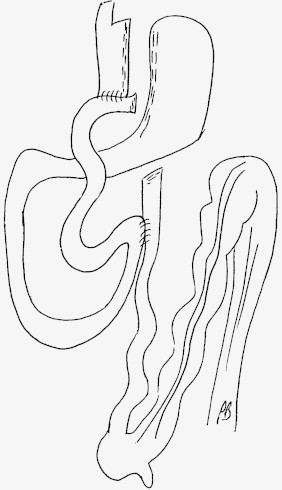
Connection in which peristalsis does not occurs in a single direction.

If a common mesentery is present, the gastric bypass is performed in the same way although the biliary-pancreatic loop comes from the patient's right (on the left on the screen) and the alimentary loop comes from the patient's left (on the right of the screen) (Fig. 5). If the angle of the Treitz ligament is not pinpointed accurately, as is always necessary, there is a risk of creating an antiperistaltic anastomosis (Fig. 6). With this anatomy, the position generally needs to be changed or trocars need to be added. It appears to us that Roux en Y gastric bypass is easier to perform if a rotation abnormality is discovered incidentally. The operation strategy can also be changed, either by not performing the intended procedure or by performing a sleeve gastrectomy. In these situations the patient must be informed of the possibility that this may occur pre-operatively. We inform patients of this as a matter of routine for all gastric bypasses. No intestinal or mesenteric pexy procedures have been proved to be useful or even safe and the bowel should be left as it is without fixation.^10^

There are only a few descriptions of gastric bypass with intestinal malrotation and these are limited to clinical cases. It is of paramount importance that the diagnosis of malrotation is made preoperatively. Therefore, the golden rule in performing a gastric bypass is to clearly visualise the angle of the Treitz ligament which allows an unknown bowel malrotation to be identified. If a common mesentery is present, the gastric bypass can still be performed. Bariatric surgeons need to be aware of these abnormalities.

## Conflict of interest statement

No.

## Funding

None.

## Ethical approval

“Written informed consent was obtained from the patient for publication of this case report and accompanying images. A copy of the written consent is available for review by the Editor-in-Chief of this journal on request”

## Author contributions

Radwan Kassir: writing; Pierre Blanc: writing; François Varlet: data collections; Christophe Breton: data collections; Patrice Lointier: data collections.
